# Timely Palliative Care: Personalizing the Process of Referral

**DOI:** 10.3390/cancers14041047

**Published:** 2022-02-18

**Authors:** David Hui, Yvonne Heung, Eduardo Bruera

**Affiliations:** Department of Palliative Care, Rehabilitation and Integrative Medicine, MD Anderson Cancer Center, Houston, TX 77030, USA; yjheung@mdanderson.org (Y.H.); ebruera@mdanderson.org (E.B.)

**Keywords:** delivery of health care, health care quality, access and evaluation, implementation, outpatient clinics, hospital, palliative care, patient-centered care, psychological distress, randomized controlled trial, referral and consultation, supportive care, symptom assessment

## Abstract

**Simple Summary:**

Timely palliative care is palliative care personalized based on patients’ needs and delivered at the optimal time and setting. It involves a systematic process to identify patients with high supportive care needs and referring these individuals to specialist palliative care in a timely manner based on standardized referral criteria. Timely palliative care brings together several important advances, including systematic symptom screening, electronic health records, and outpatient/telehealth palliative care to deliver personalized, patient-centered care towards improving patient outcomes. Empiric studies found that patients could be referred more frequently and in a timely fashion when standardized referral criteria are used. Implementation of timely palliative care at each institution requires visionary leadership, commitment of oncology teams, a robust palliative care clinic, a customized set of referral criteria and preferably an integrated electronic health record system.

**Abstract:**

Timely palliative care is a systematic process to identify patients with high supportive care needs and to refer these individuals to specialist palliative care in a timely manner based on standardized referral criteria. It requires four components: (1) routine screening of supportive care needs at oncology clinics, (2) establishment of institution-specific consensual criteria for referral, (3) a system in place to trigger a referral when patients meet criteria, and (4) availability of outpatient palliative care resources to deliver personalized, timely patient-centered care aimed at improving patient and caregiver outcomes. In this review, we discuss the conceptual underpinnings, rationale, barriers and facilitators for timely palliative care referral. Timely palliative care provides a more rational use of the scarce palliative care resource and maximizes the impact on patients who are offered the intervention. Several sets of referral criteria have been proposed to date for outpatient palliative care referral. Studies examining the use of these referral criteria consistently found that timely palliative care can lead to a greater number of referrals and earlier palliative care access than routine referral. Implementation of timely palliative care at each institution requires oncology leadership support, adequate palliative care infrastructure, integration of electronic health record and customization of referral criteria.

## 1. Introduction

Patients with cancer encounter significant supportive care needs throughout the disease trajectory, starting from the time of diagnosis [1]. These supportive care needs fluctuate with time and may include physical, psychological, social, spiritual, informational and financial concerns, often overlapping with each other, compromising patients’ quality of life. The demand for supportive care services increases with an aging patient population who often have multiple comorbid diagnoses. Moreover, there is a heightened need for supportive care in the era of novel cancer therapeutics, as patients are living longer while experiencing more chronic symptoms and adverse effects [2,3].

Over the past few decades, multiple supportive care programs have evolved to address these growing patient care needs [1]. In particular, there has been substantial development in specialist palliative care teams that provide interdisciplinary, holistic care for patients with cancer and their families [4,5,6]. Multiple randomized controlled trials have found that compared to primary palliative care provided by oncologists, early referral to specialist palliative care can improve patients’ quality of life, symptom control, mood, illness understanding, end-of-life care and survival [7,8,9,10,11,12,13]. Meta-analyses over the past 5 years have consistently reported the benefits associated with specialist palliative care [14,15,16,17,18] (Table 1). To date, the evidence on primary palliative care remains limited [19,20,21]. Thus, the focus of this article is on delivery of timely specialist palliative care.

The question is no longer whether a patient would benefit from specialist palliative care involvement, but when is the most appropriate timing of referral. This is a particularly important question given the scarcity of palliative care resources available [22]. This review focuses on the concept of timely palliative care. Specifically, we shall discuss the conceptual underpinnings, rationale, barriers, facilitators and empirical data on timely palliative care.

## 2. What Is Timely Palliative Care?

**Figure 1:**
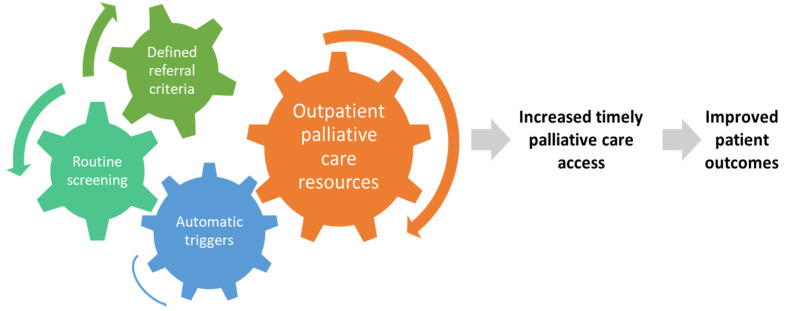
Conceptual model for timely palliative care. Timely palliative care has four key components: routine systematic screening, a defined set of referral criteria, a mechanism to trigger referral for appropriate patients, and an adequately staffed outpatient specialist palliative care clinic. The expected outcome is a greater number of patients receiving specialist palliative care and earlier timing of referral, which would lead to improved patient outcomes such as quality of life, quality of end-of-life care, and possibly survival.

Timely palliative care is a systematic process to identify patients with high supportive care needs and to refer these individuals to specialist palliative care in a timely manner based on standardized referral criteria [23]. It requires four components: (1) routine screening of supportive care needs at the oncology clinics, (2) establishment of institution-specific consensual criteria for referral, (3) having a system in place to trigger a referral when patients meet criteria, and (4) availability of outpatient palliative care resources to provide timely access (Figure 1).

## 3. How Is Timely Palliative Care Different from Early Palliative Care?

The concept of early palliative care is exemplified in the landmark Temel trial, in which patients with metastatic lung cancer were referred to palliative care within 2 months of diagnosis [24]. Although what constitutes “early” has not been established, randomized trials on early palliative care typically involved patients within 2–3 months of diagnosis of advanced diseases and had an ECOG performance status of 2 or less [7,9,10,11,25,26,27,28]. Patients referred within this timeframe are typically considered to have early palliative care involvement. Of note, patients did not need to have supportive care needs to qualify for a palliative care referral in these trials. However, a recent secondary analysis of the Zimmermann trial found that patients with higher symptom burden at baseline were more likely to derive a benefit from the palliative care intervention [29].

Due to the scarcity of palliative care resources, it is not possible to provide early palliative care for all patients with advanced disease from around the time of diagnosis [30]. Moreover, some patients may not require specialist palliative care initially due to low supportive care needs or their needs have been adequately addressed by the oncology team. In contrast to early palliative care, which initiates referral based on disease trajectory, timely palliative care is referral based on needs. Similar to early palliative care, timely palliative care is often initiated in the outpatient setting and provided to a majority of patients early in the disease trajectory.

Timely palliative care is early palliative care personalized around patients’ needs and delivered at the optimal time and setting [4]. Similar to the concept of targeted therapy, oncologists may only offer treatment for selected patients with “targetable mutations” instead of treating all patients. This approach provides a more rational use of resources, minimizes unnecessary exposure to those who may be less likely to benefit, and maximizes the impact on patients offered the intervention [29].

## 4. Rationale for Timely Palliative Care

Although specialist palliative care teams have significant expertise managing complex symptom crises that often occur in the last months of life, palliative care interventions are best provided proactively to *prevent* suffering [22,23]. Optimal timing is especially important as it allows specialist palliative care teams to more effectively introduce symptom management, provide psychological support and facilitate care planning. At MD Anderson Cancer Center, the median time from outpatient palliative care referral to death is over 12 months [31]. This allows the palliative care team to have multiple visits with patients and provide comprehensive care longitudinally.

Palliative care teams can provide a variety of non-pharmacologic and pharmacologic measures to alleviate symptoms such as pain, dyspnea and nausea when they first present. Subsequent visits can allow the palliative care team to optimize symptom control by providing therapeutic trials, active titration, proper education, longitudinal monitoring, and reinforcement of treatment adherence [32,33]. Successful symptom management not only improves quality of life, but also prevents escalation of symptoms leading to avoidable emergency room visits and hospitalizations [34,35]. Optimization of physical symptoms such as fatigue and anorexia–cachexia may also allow patients to better tolerate cancer treatments. Indeed, a higher baseline quality of life is associated with improved overall survival and progression-free survival for patients undergoing chemotherapy [36,37].

In addition to physical symptoms, timely palliative care referral allows for optimal psychological care to be delivered over time. It takes time for the palliative care team to establish trust, rapport, and explore the layers of emotional and existential concerns. Acute issues (e.g., severe pain, delirium) often need to be addressed first before chronic psychological issues can be managed. Moreover, many evidence-based interventions to treat depression and anxiety, such as counseling and exercises, require weeks and months to take effect [38,39]. In addition to patients, family caregivers benefit from building longitudinal relationships with the palliative care team so they can receive the proper education, psychologic care and resources to better support the patients throughout the disease trajectory [40,41].

Similar to symptom management and psychosocial care, serious illness conversations should start well before the last months of life because patients often require time to digest prognostic information and actively prepare for the future [42,43,44]. Palliative care teams have specialized training in communication skills to facilitate discussions around sensitive subjects such as prognostic disclosures, goals-of-care conversations and advance care planning. These discussions are longitudinal by nature and need to be timed carefully. Decisions regarding end-of-life care initiated by an oncology team are best followed by an interdisciplinary team to optimize goal-concordant care. Studies have found that early palliative care not only improves illness understanding, but also quality of end-of-life care by reducing chemotherapy use, emergency room visits, hospitalizations, intensive care units admissions in the last month of life [10,45].

## 5. Barriers and Facilitators to Timely Palliative Care

**Figure 2:**
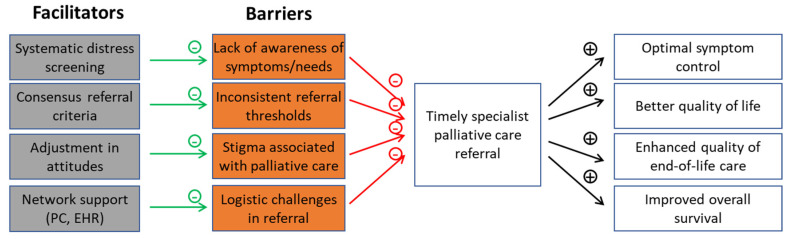
Barriers and facilitators to timely palliative care referral. Currently, there is much heterogeneity with respect to how patients with advanced cancer are being referred to specialist palliative care, resulting in low rates of referral and delayed consultations. Timely palliative care is a systematic process that is specifically designed to overcome some of the common barriers to early referral.

Despite some progress over the past decade [46], multiple barriers to timely palliative care referral exist. In this section, we will highlight several major barriers and potential solutions to overcome these challenges (Figure 2).

The lack of awareness of supportive care needs represents a major barrier to timely palliative care referral. Many symptoms remain under-reported and under-diagnosed. As a result, patients with cancer often suffer silently and are not aware of the array of supportive care options that are available. Given the high prevalence of supportive care needs among patients with advanced cancer, systematic symptom screening is essential. Several randomized controlled trials have found that routine symptom monitoring is not only associated with improved quality of life, but also longer survival. Since 2015, the Commission on Cancer has mandated distress screening, requiring oncology providers to implement standardized screening and to document an action plan [47,48,49]. Systematic symptom screening can help identify patients who may benefit from specialist referral. Routine distress screening in the oncology setting is associated with improved quality of life and survival [50,51,52,53] and represents one of the essential components of timely palliative care [54].

Stigma associated with palliative care is another major reason palliative care referrals are delayed. Currently, oncologists are the main gatekeepers for specialist palliative care referral. Specifically, many oncology teams still perceive that palliative care is for patients at the end-of-life and have reservations about initiating a referral so as not to reduce hope from patients [55,56]. This is, to a certain extent, a self-fulfilling prophecy. As palliative care continues to evolve to seeing patients earlier in the disease trajectory, more programs have changed their names to supportive/palliative care as a re-branding effort [46,57]. Oncologists are generally more open to the term “supportive care” than “palliative care” [56]. Importantly, stigma is a cultural construct; it varies across countries and institutions and changes over time. The stigma associated with “palliative care” may be reduced as timely referral to supportive/palliative care becomes the norm.

Inconsistent referral thresholds is another barrier to timely palliative care. Studies by our group and others have found significant variation in the pattern of referral among oncologists [58,59,60]. Among patients with advanced disease, solid tumor diagnosis (vs. hematological malignancies) and younger patients had greater palliative care referral [61,62]. Of interest, younger oncologists were also more likely to initiate palliative care referral, likely because they have been exposed to the principles and practice of palliative care during training [63,64]. Currently, palliative care referral often occurs in a haphazard manner, with some patients not referred at all and many others too late [65]. Results of systematic symptom screening can be used to identify patients who meet standardized referral criteria to initiate timely access to palliative care [66]. Although referral criteria have been proposed [66] (see section below), they would need to be reviewed and endorsed by both oncology and palliative care teams at each institution before successful implementation. Specifically, the thresholds need to be customized based on the level of primary palliative care and availability of downstream palliative care resource locally.

There are also logistical challenges to initiating palliative care referral. Some oncologists may not have time to review symptom scores or to initiate a referral. To overcome this barrier, a system should be put in place to match the screening data with referral criteria to identify patients most appropriate for referral. While this could be done manually, it can be quite time-consuming. The era of electronic health records may facilitate referrals by providing electronic alerts, pre-populated note templates and order sets [48]. In a Delphi survey, experts generally preferred having best practice alerts for oncologists to make the final referral decision rather than having the system automatically triggering a referral without clinician input [67].

Another system barrier to timely palliative care referral is the limited palliative care program infrastructure. A 2019 national survey of US cancer centers revealed that many palliative care programs are still under-developed due to lack of resources [46,68,69]. Although 95% of National Cancer Institute (NCI)-designated cancer centers had outpatient clinics, only 40% of non-NCI designated cancer centers reported having these services available [69]. Moreover, outpatient clinics at non-NCI designated cancer centers only operated a few days a week and were less likely to have an interdisciplinary team, which is an underpinning element of palliative care [46]. Even the larger programs had limited capacity for rapid expansion. Given these constraints, timely palliative care is best started as a pilot program in collaboration with a few oncology clinics. This would allow the program to iron out any difficulties and adjust the referral thresholds, while growing the clinic capacity in conjunction with program expansion. Institutional leadership support is also instrumental in ensuring adequate resources can be allocated to palliative care programs [70].

## 6. Referral Criteria for Outpatient Palliative Care

A 2016 systematic review of referral criteria identified 20 criteria under six themes, including physical symptoms, psychological distress, performance status, cancer trajectory, prognosis, and end-of-life care planning [71]. The lack of consensual referral criteria in the past partly explained the heterogenous pattern of referral. Several referral criteria for outpatient palliative care have been proposed since (Table 2).

Glare et al. adapted referral criteria in the National Comprehensive Cancer Network (NCCN) to develop a five-item palliative care screening tool (Table 2) [72]. The score ranges from 0 to 13, with a higher score indicating greater need. A score of ≥5 had the best predictive value.

In 2017, we conducted a Delphi study to identify consensual referral criteria for outpatient palliative care among 60 international experts [66]. Panelists reached consensus on 11 major criteria for referral (Table 2)—the presence of any criterion would be sufficient reason for a patient to be referred. Importantly, 9 of these 11 criteria were needs-based instead of time-based.

## 7. Empiric Studies on Timely Palliative Care Referral

To date, only a handful of studies have examined systematic screening to trigger palliative care referral. Although triggered referral can also apply to inpatient palliative care [73,74,75,76,77,78], the focus of our discussion is on outpatient palliative care given it is the ideal setting for timely referral.

Groenvold et al. conducted a randomized clinical trial comparing timely palliative care to standard oncologic care [79]. In this Danish study, 297 patients with advanced cancer were randomized to the two groups if they screened positive for palliative needs based on the European Organisation for Research and Treatment of Cancer Quality of Life Questionnaire (EORTC QLQ-C30). Unlike other studies that referred patients on the basis of time from diagnosis, patients were eligible if they had at least one of seven EORTC scales > 50% intensity and at least four other symptoms > 33% intensity. The investigators reported no effect on the primary outcome between the two groups (change in primary need (−4.9 points, 95% confidence interval −11.3 to +1.5 points; *p* = 0.14). However, this study had several limitations that complicated its interpretation. Specifically, the primary symptom was variable in nature. Moreover, the intervention dose was limited—44% of patients in the specialist palliative care group only had one in-person visit. The overall survival of patients included in this study was approximately 1 year, suggesting that systematic symptom screening can facilitate timely referral.

The Edmonton Symptom Assessment Scale was used for routine screening in a general oncology clinic at a safety-net hospital [48]. In the quality improvement initiative, patients who reported three or more symptoms with an intensity of 7/10 or greater would be referred to a social worker for triaging and be considered for supportive care services such as palliative care. The proportion of patients with severe symptom distress was 11%, 12% and 13% in the pre-implementation, training and post-implementation periods, respectively. The number of patients referred to palliative care increased over the three time periods (12, 20, 28%). This study provides proof-of-principle of the concept.

In a retrospective study, our group examined the referral criteria in 200 consecutive patients referred to the Supportive Care Center [31]. 170 (85%) met at least one major criteria in the international consensus with 140 (70%) meeting the “severe physical symptoms” alone. The median survival was 14 months with routine referral; these patients would be seen 2.4 months earlier if referral criteria had been systematically applied to trigger referral. Taken together, this study highlights the utility of the international consensus criteria and highlight how they could potentially be used to facilitate timely referrals.

Paiva et al. reported their experience with routine screening in two studies using a set of 16 criteria modified from the international consensus [80]. In the first study, 120 patients with advanced breast or gynecologic cancer completed the screening surveys prospectively. Only 23 (19%) were actually referred to palliative care. However, the investigators estimated that 82 (68%) more patients would be referred if the screening criteria were adopted. In addition, the timing of referral would be much earlier (451 days vs. 178 days, *p* < 0.001). In a second study, the investigators conducted a retrospective assessment of 303 patients with advanced breast or gynecologic cancer. Overall, 125 (41%) were referred. They estimated that systematic screening would refer an additional 97 (32%) patients.

In a population database study, Iqbal et al. examined 6 of the 11 major criteria in 38,851 patients with advanced lung cancer in Ontario, Canada. Of those, 15,089 (65%) were actually referred to palliative care before death. The authors reported that, 23,199 (82.4%) of patients would be eligible for palliative care based on the criteria, with a median duration of 56 days between eligibility to either actual receipt of palliative care or last follow-up/death [81]. Although the palliative care referral was approximately 2 months earlier, it was less than other studies.

Kim et al. conducted a quality improvement project to screen for palliative care needs among patients with glioblastoma at Duke Cancer Institute [82]. The NCCN screening tool was administered by advanced practice providers at the neurooncologist clinic to 294 of 530 eligible patients (56%) and 27 (9%) of those who completed the screening screened positive (i.e., score ≥ 5). 17/27 (63%) had a discussion on palliative care referral and 12 (71%) were actually referred. Overall, 10/27 (37%) patients did not have a discussion on palliative care referral because of a focus on treatment or attending physician disagreement.

Cancer Care Ontario reported its experience with the “Integrating Early Palliative Care into Routine Practice for Patients with Cancer” (INTEGRATE) program in a population-based retrospective cohort study [83]. In this intervention, a patient was considered to be appropriate for specialist palliative care if the clinician agreed that they would not be surprised if the patient dies within 6–12 months. Overall, 1185 patients from four cancer centers and four primary care teams were included in the intervention group and were compared against 1185 patients who received usual care. In a propensity-score matched analysis, the intervention group had significantly greater palliative care referral (80% vs. 62%; HR 1.69) and higher proportions of home care (81% vs. 55%; HR 2.07). Hospitalizations that were not primarily focused on palliative care (HR 1.42) and unplanned emergency department visits for non-palliative care purposes (HR 1.47) also increased in the intervention group. Quality of life outcomes were not documented.

Taken together, these studies highlight that timely palliative care referrals could lead to a greater number of referrals and earlier palliative care access than routine referrals. More studies have examined the international consensus criteria to date than other criteria. However, few of these studies involved actual referral because of the lack of palliative care clinic resources. Further research is needed to customize referral criteria for different institutions and to integrate systematic screening and referral orders into the electronic health record.

## 8. Implementation of Timely Palliative Care in the Real World

Although the four key components (screening, criteria, trigger, and palliative care) are essential for timely palliative care referral, successful implementation of this model on a wider scale would require us to consider many potential political, cultural, administrative, logistical and financial challenges. Specifically, it is critical to secure institutional buy-in from clinicians and leadership, administrative support for program coordination, and adequate resources for long term sustainability. Without adequate preparations, full scale institution-wide implementation is unlikely to be successful, especially at larger institutions. Thus, we recommend starting with quality improvement pilot projects involving a few interested ‘palliphilic’ oncologists first. This would allow time to properly consult all involved parties, identify appropriate referral thresholds, develop the necessary structure and processes, provide staff education, and document key outcomes. The data gathered will allow program refinements and justify further expansion, if appropriate.

Demonstration projects to share the success and challenges of implementing timely palliative care referral are needed. The RE-AIM framework represents a structured approach to translating research to practice and may be used for timely palliative care [84,85]. Key domains of this framework include examining the program’s reach (identifying and approaching target population), effectiveness (documenting outcomes), adoption (understanding providers’ perspectives and organizational challenges), implementation (adherence to intervention) and maintenance (sustainability) [86,87]. A full discussion of implementation science is beyond the scope of this review, and readers are encouraged to refer to other resources [84,85].

## 9. Commonly Asked Questions Regarding Timely Palliative Care Referral

**Oncologists are currently referring patients based on needs. How is that different from timely palliative care?** Although oncologists currently refer patients based on perceived needs, there is tremendous heterogeneity in the referral process because of a lack of systemic symptom screening and standardized referral thresholds. In contrast, timely palliative care referral means that patients’ needs are universally assessed and the referral process is standardized and streamlined, allowing referrals to occur much more frequently and earlier than the current approach.

**What is the role of primary palliative care in this model of timely palliative care?** Studies have found that specialist palliative care, when added on top of primary palliative care delivered by non-palliative care clinicians, can improved outcomes compared to primary palliative care alone. In contrast, the evidence to support primary palliative care alone remains limited. Being in the front lines, primary palliative care has an important role providing basic supportive care in a timely fashion and identifying patients appropriate for specialist palliative care referral [14,15,16,17,18]. Patients whose supportive care needs are adequately addressed by primary palliative care may not require a specialist palliative care referral. Ultimately, the threshold for referral needs to be carefully defined at each institution.

**Should timely palliative care be limited to the outpatient setting?** Outpatient is the main setting for ambulatory patients [4]; thus, outpatient palliative care clinics are is the most appropriate setting for timely palliative care. Studies have found that outpatient palliative care consultations are associated with lower rates of unplanned emergency room visits and hospitalizations at the end-of-life compared to inpatient palliative care consultations [34,35]. Triggered referral may also be instituted in the inpatient setting to streamline palliative care access [73,76]; however, ideally patients have had outpatient encounters already.

**Do the referral criteria need to be followed strictly?** The referral criteria in Table 2 provide a starting point for some key triggers to an outpatient palliative care referral. However, the specific criteria set and cutoff should be tailored to each institution based on discussions between the oncology and palliative care teams. They should then be pilot tested and revised further before wider-scale implementation.

**What is the role of electronic health records in timely palliative care?** The electronic health record may be involved in many of the steps along the referral process, including (1) administration of electronic patient reported outcomes for screening, (2) displaying the supportive care needs for healthcare providers, (3) matching the needs against referral criteria, (4) best practice alerts for health professionals if a patient is found to be appropriate for palliative care, (5) facilitating the referral order, and (6) providing longitudinal monitoring of key metrics [48,88]. As shown in the pilot studies above, any of these steps could be conducted manually as well; however, electronic interface would clearly be advantageous for wide-scale implementation.

**Can this process of timely palliative care referral be completely automated?** Although triggered referral could theoretically take place independent of clinician input, it is important to highlight that the computer should be considered as a tool to augment, rather than replace, clinician judgement [67]. Patients would need to be informed about the role of supportive/palliative care and would benefit from verbal endorsement by their oncologists prior to referral.

**Is there a time cutoff for timely palliative care?** The right time for each patient is personalized based on their needs. Although there is no specific time cutoff, a vast majority of patients are expected to be referred 6–18 months before death based on the international consensus criteria [31,80].

**What are future research directions for timely palliative care?** Randomized clinical trials can be used to examine the concept of timely palliative care. By focusing the study population on those identified to have supportive care needs, the effect size with palliative care intervention is expected to be greater. Demonstration projects and studies examining various aspects of program implementation would provide useful insights on how timely palliative care can be achieved in different settings.

## 10. Conclusions

Timely palliative care is early palliative care personalized based on patients’ needs. It involves a systematic process to identify patients with high supportive care needs and referring these individuals to specialist palliative care in a timely manner based on standardized referral criteria. Timely palliative care brings together several important advances, including systematic symptom screening, electronic health records, and outpatient/telehealth palliative care to deliver personalized, patient-centered care aimed towards improving patient outcomes. Empiric studies found that patients could be referred more frequently and in a timely fashion when standardized referral criteria are used. Implementation of timely palliative care at each institution requires visionary leadership, commitment of oncology teams, a robust palliative care clinic, a customized set of referral criteria and an integrated electronic health record system. Further research is needed to examine the impact of timely palliative care on patients, caregivers and health system outcomes.

## Figures and Tables

**Table 1:** Meta-analyses on the outcomes of specialist palliative care for patients with cancer.

	Setting	No. of Studies	No. of Patients	Quality of Life SMD (95% CI)	Symptoms SMD (95% CI)	Mood SMD (95% CI)	Survival HR (95% CI)
Kavalieratos et al. 2016 [16]	IP/OP	11	1670	0.12 (−0.2, 0.27)	−0.14 (−0.39, 0.10)		0.82 (0.60, 1.13)
Gartner et al. 2017 [14]	IP/OP	5	828	0.20 (0.01, 0.38)	−0.21 (−1.35, 0.94)		
	OP (early only)	2	388	0.33 (0.05, 0.61)			
Haun et al. 2017 [15]	OP	7	1614	0.27 (0.15, 0.38)	−0.23 (−0.35, −0.10)	−0.11 (−0.26, 0.03)	0.85 (0.56, 1.28)
Heorger et al. 2019 [17]	OP	8	2092	0.18 (0.09, 0.28)			1y: 14.1% (6.5%, 21.7%)
Fulton et al. 2019 [18]	OP	10	2385	0.24 (0.13, 0.35)	−0.17 (−0.45, 0.11)	−0.09 (−0.32, 0.13)	0.84 (0.61, 1.18)

Abbreviations: CI, confidence interval; HR, hazard ratio; IP, inpatient; OP, outpatient; SMD, standardized mean difference.

**Table 2:** Referral criteria for outpatient palliative care.

International Consensus [66]	NCCN Referral Criteria [72]
**Development**	**Development**
International Delphi consensus panel	Modified based on NCCN recommendations
Pilot tested in outpatient settings	Pilot tested in outpatient and inpatient settings
**Criteria**	**Criteria**
Severe physical symptoms (e.g., pain, dyspnea or nausea scored 7–10 on a ten-point scale) Severe emotional symptoms (e.g., depression or anxiety scored 7–10 on a ten-point scale) Request for hastened death Spiritual or existential crisis Assistance with decision making or care planning Patient request Delirium Brain or leptomeningeal metastases Spinal cord compression or cauda equina Within 3 months of diagnosis of advanced or incurable cancer for patients with median survival of 1 year or less Diagnosis of advanced cancer with progressive disease despite second-line systemic therapy (incurable)	Presence of metastatic or locally advanced cancer [2 points] Functional status score, according to ECOG performance status score [0–4 points] Presence of one or more serious complications of advanced cancer usually associated with a prognosis of <12 months (e.g., brain metastases, hypercalcemia, delirium, spinal cord compression, cachexia) [1 point] Presence of one or more serious comorbid diseases also associated with poor prognosis (e.g., moderate-severe COPD or CHF, dementia, AIDS, end stage renal failure, end stage liver cirrhosis) [1 point] Presence of palliative care problems Symptoms uncontrolled by standard approaches [1 point] Moderate to severe distress in patient or family, related to cancer diagnosis or therapy [1 point] Patient/family concerns about course of disease and decision making [1 point] Patient/family requests palliative care consult [1 point] Team needs assistance with complex decision-making or determining goals of care [1 point]
**Suggested threshold for referral**	**Suggested threshold for referral**
Presence of any criterion above would indicate a patient is appropriate for a specialist palliative care referral	A total score of ≥5 indicates a patient is appropriate for a specialist palliative care referral

Abbreviations: AIDS—advanced immunodeficiency syndrome; CHF—congestive heart failure; COPD—chronic obstructive pulmonary disease; ECOG—Eastern Cooperative Oncology Group.
